# Trajectory of Heart Failure Severity After Acute Myocardial Infarction

**DOI:** 10.1016/j.jacasi.2026.04.006

**Published:** 2026-05-30

**Authors:** Yuichi Saito, Yoshiyuki Ohnaga, Naoto Mori, Osamu Hashimoto, Takaaki Matsuoka, Masahiro Suzuki, Satoshi Tokimasa, Norikiyo Oka, Shinya Ichihara, Takanori Sato, Yoshihide Fujimoto, Hideki Kitahara, Yoshio Kobayashi

**Affiliations:** a Department of Cardiovascular Medicine, Chiba University Hospital, Chiba, Japan; b Department of Cardiology, Chiba Aoba Municipal Hospital, Chiba, Japan; c Department of Cardiology, Chiba Emergency and Psychiatric Medical Center, Chiba, Japan; d Department of Cardiology, Japanese Red Cross Narita Hospital, Narita, Japan; e Department of Cardiology, Chibaken Saiseikai Narashino Hospital, Narashino, Japan; f Department of Cardiology, Chiba Rosai Hospital, Ichihara, Chiba, Japan; g Department of Cardiology, Funabashi Municipal Medical Center, Funabashi, Japan; h Department of Cardiovascular Medicine, Asahi General Hospital, Asahi, Japan; i Department of Cardiovascular Medicine, Eastern Chiba Medical Center, Togane, Japan; j Department of Cardiology, International University of Health and Welfare Narita Hospital, Narita, Japan

**Keywords:** acute myocardial infarction, heart failure, natriuretic peptide

Even in the current era, heart failure (HF) events after acute myocardial infarction (AMI) remain a major consequence.^1^^,^^2^ In patients with suspected and established HF, the guidelines recommend evaluating natriuretic peptides, such as B-type natriuretic peptide (BNP) and N-terminal pro–B-type of BNP (NT-proBNP).^3^ Although the dynamic changes and prognostic impact of BNP and NT-proBNP in the early phase after AMI (days to weeks) have been established,^4^, ^5^, ^6^ the mid- to long-term trajectory of these biomarkers beyond the first several months remains unclear.

This was a prospective, multicenter, observational study at 10 hospitals in Chiba prefecture, Japan (AMI and HF registry in Chiba). Patients with AMI undergoing primary percutaneous coronary intervention (PCI) within 48 hours were eligible when complicated by HF during hospitalization, assessed with BNP >100 pg/mL and/or NT-proBNP ≥400 pg/mL,^7^ irrespective of clinical signs of congestion. Major exclusion criteria included maintenance hemodialysis, impaired renal function (estimated glomerular filtration rate <30 mL/min/1.73 m^2^), NT-proBNP <400 pg/mL during hospitalization, failed primary PCI, and in-hospital death. The present study was centrally approved by the ethics committee of Chiba University Hospital, and all patients provided written informed consent. This study was registered in the University Hospital Medical Information Network (UMIN000046004). Patients were followed up at 1, 3, and 12 months after AMI. Treatment strategies were left to local physicians, but optimal medical therapy was encouraged. The primary endpoint was a trajectory of NT-proBNP levels after AMI at each time point. HF-related events and major adverse cardiac events (MACE) were defined as a composite of cardiovascular death and HF hospitalization, cardiovascular death, AMI, and ischemic stroke, respectively. All statistical analyses were performed using R software version 4.3.1 (The R Foundation for Statistical Computing) and SAS software version 8.3 (SAS Institute). Log-transformed NT-proBNP levels at each time point were analyzed using a linear mixed model with a random intercept with Bonferroni adjustment for multiple comparisons. The incidence of outcomes was estimated using the Kaplan-Meier method with 95% CIs. The receiver operating characteristic curve analysis was performed for HF-related events and MACE, and the best cut-off value was established based on Youden’s index. *P* <0.05 was considered statistically significant.

Between November 2021 and March 2024, 1,980 patients with AMI underwent primary PCI, among whom 930 were excluded. The most common exclusion criterion was NT-proBNP <400 pg/mL, followed by in-hospital death and renal impairment. Thus, 1,050 patients with AMI and HF were included in the present study. The patterns of age and sex were similar between those patients included and excluded. Of the 1,050 patients, the mean age was 69.7 ± 11.7 years, men accounted for 78.0% (n = 819), and the prevalence of hypertension, diabetes, dyslipidemia, and current smoking was 75.0% (n = 788), 41.9% (n = 440), 69.2% (n = 727), and 35.0% (n = 367), respectively. Patients presented with ST-segment elevation myocardial infarction and cardiogenic shock in 747 (71.1%) and 105 (10.0%), respectively. The mean left ventricular ejection fraction was 51.3% ± 10.5%, and the mean estimated glomerular filtration rate was 62.5 ± 18.8 mL/min/1.73 m^2^. Primary PCI was performed with radial artery approach, intracoronary imaging, and drug-eluting stents in 968 (92.2%), 1,043 (99.3%), and 979 (93.2%) of 1,050 patients, respectively. Angiotensin-converting enzyme inhibitors, angiotensin II receptor blockers, or angiotensin receptor-neprilysin inhibitors, β-blockers, mineralocorticoid receptor antagonists, and sodium-glucose cotransporter 2 inhibitors were prescribed in 886 (84.4%), 807 (76.9%), 357 (34.0%), and 389 (37.0%) patients at discharge, respectively.

The participants were followed up with a median of 365 (Q1-Q3: 341-380) days. The 12-month incidence of HF-related events and MACE was estimated as 3.7% (95% CI: 2.5% to 4.9%) and 4.4% (95% CI: 3.0%–5.7%), with cardiovascular death in 1.0% (95% CI: 0.3%–1.6%), HF hospitalization in 3.2% (95% CI: 2.1%–4.4%), recurrent AMI in 2.7% (95% CI: 1.6%–3.8%), and ischemic stroke in 0.8% (95% CI: 0.2%–1.4%), respectively. The trajectory of NT-proBNP after discharge, the primary endpoint, is illustrated in Figure 1. The declining trend is also depicted as relative changes to baseline values (at discharge) (Figure 1). The receiver operating characteristic curve analysis showed that NT-proBNP levels at discharge were predictive of HF-related events after discharge (area under the curve 0.740; 95% CI: 0.656–0.824, best cut-off value 1,831 pg/mL, *P* < 0.001), while it did not predict MACE (area under the curve 0.523; 95% CI: 0.436–0.610, *P* = 0.625).

**Figure 1:**
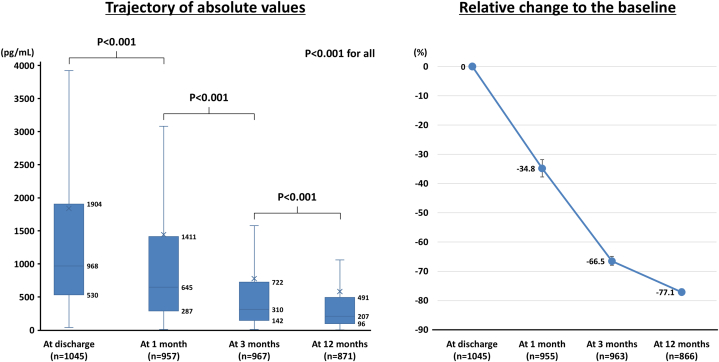
Trajectories of NT-proBNP After AMI Absolute levels of N-terminal pro–B-type natriuretic peptide (NT-proBNP) consistently declined during the 12-month follow-up period after acute myocardial infarction (AMI) (left). Box plots represent the median, IQR, and range. Relative changes from baseline were calculated as the percentage change in estimated geometric means derived from a linear mixed model with a random intercept for each patient, using log-transformed NT-proBNP as the dependent variable (right). Error bars represent 95% CIs. *P* values were derived from the linear mixed model with Bonferroni adjustment for multiple comparisons (*P* <0.001 for all pairwise comparisons).

The present prospective, multicenter, observational registry study demonstrated that among patients with AMI undergoing successful primary PCI, more than 80% had an elevated NT-proBNP level (≥400 pg/mL) during hospitalization. NT-proBNP levels consistently declined during the 12-month follow-up period, and the higher levels of NT-proBNP at discharge were significantly associated with an increased risk of HF-related events after discharge.

Early identification and management of HF after AMI is clinically relevant, and NT-proBNP is considered a robust biomarker.^8^ While numerous studies have reported the change and prognostic value of natriuretic peptides in the early phase after AMI,^4^, ^5^, ^6^ the longitudinal data beyond the first several months remain limited.^9^ We believe that the long-term trajectory of NT-proBNP levels observed in the present study can be a benchmark for healthcare professionals and future research. Our findings also suggest that repeated NT-proBNP measurements are useful for risk stratification in patients with AMI and potential HF. Abnormal NT-proBNP levels after AMI, such as elevation at discharge, an increase from discharge to 1 month, and no decline during the 12-month follow-up, may indicate higher HF risks and warrant intensive treatment strategies. Although early pharmacological intervention can improve HF outcomes, as reported in the STRONG-HF trial,^10^ further studies are needed to elucidate the safety and efficacy of such treatment strategies with anti-HF medications on natriuretic peptide levels and HF events in patients with AMI. In the present study, we primarily evaluated the long-term trajectory of NT-proBNP after AMI, while this study was underpowered for clinical outcome events. Additionally, patients were eligible if they had elevated natriuretic peptide levels without clinical signs of congestion, irrespective of atrial fibrillation status.

Nonetheless, we believe that our results showed absolute values and relative changes in NT-proBNP levels after AMI in contemporary practice as a reference standard and reinforce the importance of repeated natriuretic peptide assessment in patients with AMI.

## Funding Support and Author Disclosures

This work was supported by the Japan Society for the Promotion of Science Grant in Aid for Scientific Research (KAKENHI) grants (grant number 24K13335 to Dr Yuichi Saito). Dr Saito has received speaker fees from Daiichi-Sankyo and Novartis. Dr Kobayashi has received lecture fees from Amgen, Novartis, Medtronic Japan, and Daiichi-Sankyo and research grants from Abbott Medical Japan, Win International, Nipro, Kaneka Medics, and OrbusNeich Medical. All other authors have reported that they have no relationships relevant to the contents of this paper to disclose.
